# Le nouveau vaccin antipoliomyélitique oral : Un tournant décisif pour le programme d’éradication ?

**DOI:** 10.48327/mtsi.2021.191

**Published:** 2021-12-20

**Authors:** Maël BESSAUD

**Affiliations:** Institut Pasteur, Unité pathogenèse & populations virales, 25-28, rue du Dr Roux, 75015 Paris, France

**Keywords:** Poliomyélite, Poliovirus, VDPV, *Vaccine-derived poliovirus*, Vaccin polio oral, Éradication, Poliomyelitis, Poliovirus, VDPV, Vaccine-derived poliovirus, Oral polio vaccine, Eradication

## Abstract

Lancé en 1988, le programme d’éradication de la poliomyélite vise à éradiquer les poliovirus, agents étiologiques de la maladie. Coordonné par l'Organisation mondiale de la santé, le programme repose sur des campagnes de vaccination de routine ciblant les enfants et sur la surveillance active de la circulation des virus. Il a permis l’éradication de deux des trois sérotypes de poliovirus sauvages et a circonscrit la circulation du sérotype restant à deux pays seulement.

Deux vaccins antipoliomyélitiques existent: le vaccin injectable et le vaccin oral. Si les deux vaccins offrent une protection similaire contre la maladie, seul le second est capable de bloquer la transmission des poliovirus. Le vaccin oral est donc indispensable pour endiguer les poliovirus et, finalement, les éradiquer. Dans certains contextes où la couverture vaccinale est faible, les souches atténuées qui composent le vaccin oral peuvent circuler durant des mois et recouvrer un phénotype pathogène par dérive génétique. Afin d’éviter ce phénomène, une nouvelle souche vaccinale a été développée par génie génétique: elle a été conçue pour être aussi immunogène que la souche vaccinale historique mais beaucoup plus stable génétiquement afin d’éviter la perte des déterminants génétiques de son atténuation. Après une phase d’évaluation *in vitro* et des essais cliniques visant à confirmer ses propriétés, la nouvelle souche a été mise en œuvre dans plusieurs pays africains et au Tadjikistan en 2021.

## Abstract (en)

Launched in 1988, the Global Polio Eradication Initiative (GPEI) aims to eradicate polioviruses, which are the etiologic agents of poliomyelitis. Coordinated by the World Health Organization, this program relies on two pillars: mass vaccination campaigns that target children and active surveillance of the virus circulation. The GPEI has led to the eradication of two out of three serotypes of wild polioviruses and to the containment of the last serotype in two countries.

Two polio vaccines exist: the injectable vaccine and the oral one. Both induce an efficient protection against poliomyelitis, but only the oral vaccine is able to stop poliovirus transmission chains. Therefore, the oral vaccine is essential to contain polioviruses and, finally, to eradicate them. In some contexts where the vaccine coverage is not sufficient, the attenuated strains contained in the oral vaccine can circulate for months and recover a pathogenic phenotype through genetic drift. In order to prevent this phenomenon, a new vaccine strain has been developed through genetic engineering: it has been designed to be as immunogenic as the historical vaccine strain, but more genetically stable to prevent the loss of its attenuation determinants. After being evaluated in vitro and through clinical trials, the novel strain has been rolled out in several African countries and in Tajikistan in 2021.

## La poliomyélite et le programme mondial d’éradication

**Figure 1:**
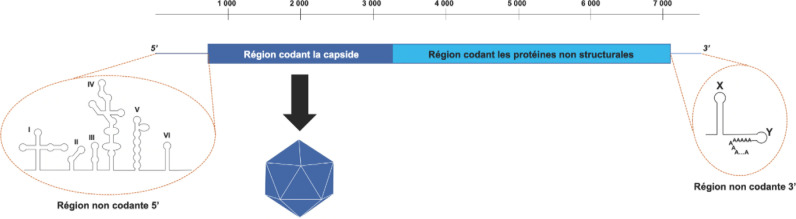
Représentation schématique du génome des poliovirus. Le génome est composé d'une molécule unique d'ARN monocaténaire d'environ 7 500 nucléotides. Il sert directement d'ARN messager lors de l'infection. Il contient un long cadre de lecture qui code une polyprotéine comportant les protéines structurales qui composeront la capside du virion et les protéines non structurales indispensables au cycle viral. De part et d'autre de ce cadre de lecture, deux régions non codantes possèdent des structures tridimensionnelles (I à VI, X et Y) indispensables à la traduction et la réplication du génome. L'extrémité 3’ du génome est polyadénylée Scheme of the poliovirus genome. The genome is made of a unique single-stranded RNA of about 7,500 nucleotides in length. It acts as a messenger RNA during the infection. It contains a long open reading frame that encodes a polyprotein from which the structural proteins that form the virus capsid and the non-structural proteins involved in the virus cycle are generated by cleavages. On either side of the open reading frame, two untranslated regions bear three-dimensional structures (I-VI, X and Y) that are crucial for the translation and the replication of the genome. The 3’ genome extremity is poly-adenylated

La poliomyélite est une maladie caractérisée par une perte de tonus musculaire d’étendue variable. La maladie est due aux poliovirus appartenant à la famille des *Picornaviridae,* genre *Enterovirus.* Il existe trois sérotypes de poliovirus qui tous peuvent déclencher la maladie. Les particules virales, de forme icosaédrique, sont non enveloppées et peuvent rester infectieuses relativement longtemps dans l'environnement. Elles contiennent une molécule unique d'ARN monocaténaire qui constitue le génome viral (Fig. 1). Le site de réplication principal des poliovirus se situe dans l'intestin, et les particules virales produites sont excrétées dans les selles. La transmission se fait ainsi par voie féco-orale en particulier *via* l'eau ou les aliments souillés par des excréments humains.

Dans la plupart des cas, l'infection par un poliovirus est asymptomatique ou ne déclenche qu'un syndrome fébrile sans gravité. Dans de rares cas toutefois, le virus atteint le système nerveux central dans lequel sa réplication peut être à l'origine de symptômes sévères. Ainsi, sa réplication dans les motoneurones de la corne antérieure de la moelle épinière entraîne leur destruction; le résultat est une faiblesse musculaire, voire une paralysie totale des muscles commandés par les neurones détruits. La localisation des symptômes musculaires dépend de l’étage médullaire atteint: la paralysie peut toucher les membres, la face, les muscles oculomoteurs, les muscles impliqués dans la déglutition, etc. L'atteinte de fibres nerveuses commandant le diaphragme conduit à une paralysie diaphragmatique qui nécessite un recours à la ventilation mécanique pour éviter l'asphyxie du sujet atteint. La destruction des neurones moteurs étant irréversible, les survivants conservent souvent des séquelles lourdes fortement handicapantes.

Lancée en 1988, l'Initiative mondiale pour l’éradication de la polio vise à éradiquer les poliovirus, c'est-à-dire à stopper définitivement la circulation des trois sérotypes à l’échelle du globe. Leur éradication est envisageable puisqu'il n'existe aucun réservoir animal de ces virus. La stratégie du programme consiste à établir une immunité collective la plus élevée possible par le biais de campagnes de vaccination ciblant les enfants de moins de cinq ans. En parallèle, un réseau de 150 laboratoires coordonné par l'OMS assure la surveillance de la circulation des poliovirus afin de déclencher des ripostes vaccinales ciblées dans le but d'endiguer le plus rapidement possible les épidémies. Le programme d’éradication a permis un net recul de la maladie: estimé à plusieurs centaines de milliers à la fin des années 1980, le nombre annuel de cas de poliomyélite est tombé à quelques centaines à la fin des années 2010. Dans le même temps, l'aire de circulation des virus sauvages a été réduite de façon drastique: alors qu'ils circulaient sur tous les continents au début du programme, ils sont aujourd'hui circonscrits dans deux pays: le Pakistan et l'Afghanistan. Enfin, deux des trois sérotypes ont été éradiqués: aucun virus sauvage de type 2 n'a été détecté depuis 1999, et le dernier isolement d'un poliovirus sauvage de type 3 remonte à 2015.

## Le vaccin polio oral d'albert sabin

La vaccination repose sur deux vaccins développés dans les années 1950-1960: le vaccin polio injectable (VPI) et le vaccin polio oral (VPO). Le VPI contient des virions des trois sérotypes produits en cultures cellulaires puis inactivés par traitement au formaldéhyde. Le VPO contient trois souches vivantes atténuées, appelées souches Sabin d'après le nom de leur inventeur, Albert Sabin.

**Figure 2:**
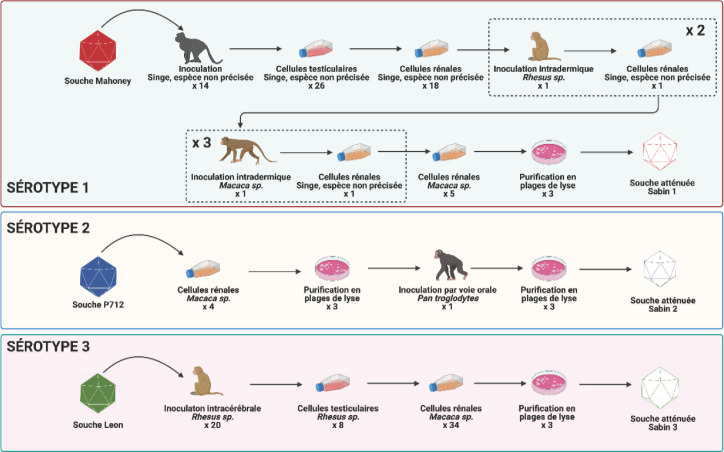
Processus ayant permis d'obtenir les 3 souches atténuées Sabin à partir d'une souche sauvage de chacun des sérotypes. Figure réalisée en utilisant le logiciel BioRender (https://biorender.com) Process used to obtain the 3 attenuated strains from one wild strain of each serotype. Figure created using BioRender software (https://biorender.com)

**Figure 3:**
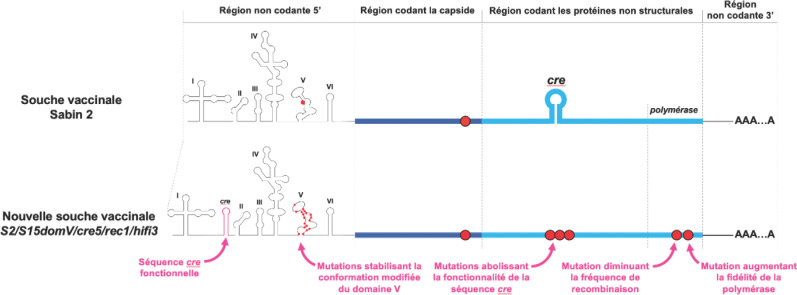
Représentation schématique du génome des souches atténuées Sabin 2 et S2/S15domV/cre5/rec1/hifi3. L'atténuation de la souche Sabin 2 ne repose que sur deux positions nucléotidiques (indiquées par un disque rouge) dont celle située dans le domaine V de la région non codante 5’ est la plus importante. Le génome de la souche S2/S15domV/cre5/rec1/hifi3 possède des mutations supplémentaires dans ce domaine afin d'en stabiliser la conformation tridimensionnelle qui est responsable de l'atténuation Scheme of the genomes of the attenuated Sabin 2 and S2/S15domV/cre5/rec1/hifi3 strains. Sabin 2 attenuation relies on two nucleotides only (red circles), among which the major one is located in the V domain of the 5’ untranslated region. In the S2/S15domV/cre5/rec1/hifi3 genome, additional mutations were introduced in this domain to stabilize its three-dimensional conformation, which is responsible for the attenuated phenotype

Ces dernières ont été développées selon la technique d'atténuation par passages successifs des virus, une technique empirique qui repose sur le fait que l'adaptation d'un virus à un environnement différant substantiellement de son environnement naturel s'accompagne souvent d'une diminution de sa capacité à se répliquer dans celui-ci [10]. Le virus que l'on souhaite atténuer est inoculé à des animaux et/ou cultivé *in vitro* jusqu’à perdre son pouvoir pathogène chez l'Homme. Plusieurs vaccins ont été développés selon cette stratégie, tels ceux contre la fièvre jaune ou la varicelle [16]. Dans le cas des poliovirus, les souches vaccinales ont été obtenues par des schémas complexes de cultures successives dans différents types de cellules de singes, d'inoculations à des singes de différentes espèces et de purification par la technique des plages de lyse (Fig. 2) [20]. Les souches vaccinales Sabin conservent la capacité à se répliquer dans l'intestin des personnes auxquelles elles sont administrées, mais ne peuvent pas se répliquer dans les cellules nerveuses. Leur atténuation ne repose que sur quelques modifications de la séquence nucléotidique du génome viral [3]. Pour les trois souches vaccinales, le déterminant majeur de l'atténuation est une mutation unique située dans le domaine V de la région non codante 5’ du génome (Fig. 3). Cette mutation induit un changement de la conformation du domaine V qui rend le virus inapte à se répliquer efficacement dans les cellules nerveuses.

Les avantages et inconvénients respectifs du VPI et du VPO ont conduit les États à privilégier l'un ou l'autre selon le contexte. Le VPI offre l'avantage d'une innocuité complète tandis que le VPO, comme tous les vaccins vivants, peut induire des effets indésirables chez les personnes immunodéprimées. Par ailleurs, dans de très rares cas, les souches vaccinales peuvent atteindre le système nerveux et induire une poliomyélite suite à la réversion des déterminants nucléotidiques de l'atténuation lors de leur réplication dans l'intestin du vacciné [19]. La fréquence de tels cas de poliomyélite associée à la vaccination est estimée à un pour 2,7 millions de doses. Toutefois, le VPO présente de nombreux avantages par rapport au VPI: en premier lieu, son coût de production est inférieur; de plus, ne requérant pas d'injection, il peut être administré par du personnel non médical; enfin, son conditionnement en flacons multidoses permet de vacciner rapidement de nombreux enfants les uns à la suite des autres et de procéder à des campagnes de « ratissage » durant lesquelles tous les enfants rencontrés par les vaccinateurs reçoivent une dose de vaccin. Ces caractéristiques ont fait du VPO le vaccin de choix dans de nombreux pays où le système sanitaire n'est pas suffisamment performant pour garantir la vaccination de tous les enfants par le VPI. De surcroît, le VPO possède un avantage décisif sur le VPI: en se multipliant dans l'intestin des personnes vaccinées, les souches qui composent le VPO induisent une immunité mucosale qui limite la réinfection des vaccinés par une souche de poliovirus sauvage. Le VPI quant à lui induit une immunité sérique qui prévient l'accès des poliovirus au système nerveux central mais il n'induit qu'une faible immunité intestinale; celle-ci est insuffisante pour empêcher la réplication d'un poliovirus sauvage en cas d'exposition. Les personnes vaccinées avec le VPI sont donc protégées contre la maladie mais restent susceptibles de transmettre le virus sauvage. Seul le VPO étant capable de bloquer la transmission du virus, son utilisation est incontournable pour stopper les épidémies. Ainsi, une souche sauvage de type 1 (probablement importée du Pakistan) a pu circuler en Israël durant un an (2013-2014) malgré une excellente couverture vaccinale [4]. Le pays ayant abandonné le VPO quelques années plus tôt, tous les enfants nés après 2005 n'avaient été immunisés que par le VPI et constituaient ainsi une population susceptible de permettre la circulation du virus tout en étant protégée de la maladie. La circulation du virus n'a pu être stoppée que par la réintroduction du VPO, qui a été administré à plus de la moitié des enfants nés après 2005 [11].

## Le problème des souches dérivées du vaccin

**Figure 4:**
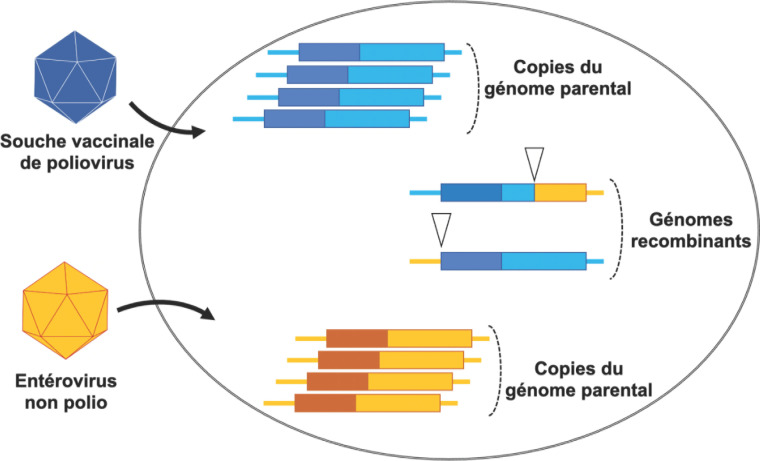
Événements de recombinaison entre un poliovirus et un entérovirus non polio infectant la même cellule. La réplication simultanée du génome des deux virus conduit à l'apparition de génomes chimériques. Ceux-ci sont constitués de séquences provenant des deux génomes parentaux. Les points de recombinaison (indiqués ici par des triangles) se trouvent généralement à la fin de la région non codante 5’ ou au sein de la partie du génome codant les protéines non structurales. La succession d’événements de recombinaison impliquant de nombreux entérovirus génère en permanence de nouveaux génomes viraux. Lors de leur circulation au sein d'une population insuffisamment vaccinée, les souches vaccinales de poliovirus participent elles aussi à ces événements de recombinaison. Dans certains génomes de VDPV, seule la séquence codant la capside provient d'une souche Sabin, le reste du génome provenant d'entérovirus non polio Recombination events between a poliovirus and a non-polio enterovirus infecting the same cell. The concomitant replication of the genomes of both viruses generates chimeric genomes made of sequences coming from the two parental viruses. The breakpoints (indicated by triangles) are generally located at the very end of the 5’ untranslated region and within the genomic region that encodes the non-structural proteins. Successive recombination events between numerous enteroviruses continuously generate new genomes. When circulating among populations with insufficient vaccine coverage, the poliovirus vaccine strains are involved in these recombination events. In some VDPV genomes, only the capsid-encoding region comes from the poliovirus vaccine strain while the rest of the genome is from non-polio enteroviruses

Le VPO présente un défaut majeur du fait de l'instabilité génétique des souches qui le composent. Lorsque la couverture vaccinale est trop faible, les souches vaccinales peuvent être transmises d'une personne non immunisée à une autre durant de longues périodes. La circulation des souches s'accompagne d'une accumulation de mutations dans leur génome. De grandes régions de celui-ci peuvent même être brusquement modifiées par des événements de recombinaison. Ces derniers se produisent lorsque deux virus se répliquent simultanément dans la même cellule; ils aboutissent à l'apparition de génomes chimériques constitués de séquences provenant des deux virus parentaux (Fig. 4). De tels événements sont très fréquents au sein des écosystèmes que constituent les entérovirus [17]. Les poliovirus en général et les souches vaccinales en particulier peuvent recombiner avec de nombreux entérovirus de l'espèce C pour donner des génomes fonctionnels [6]. Les mutations et les événements de recombinaison peuvent entraîner la perte des marqueurs d'atténuation et donner ainsi naissance à des souches dérivées du vaccin (VDPV, pour *vaccine-derived polioviruses)* aussi neurovirulentes que les souches sauvages.

L'implication de VDPV dans des épidémies de poliomyélite n'a été découverte qu'au début des années 2000 lors d'une épidémie à Saint-Domingue. Depuis, de nombreuses épidémies ont été attribuées à des VDPV dans des régions où le VPO était utilisé et où il était difficile d'atteindre et de maintenir une couverture vaccinale élevée [5]. Tandis que l'aire de circulation des virus sauvages et le nombre de cas de poliomyélite dont ils étaient responsables diminuaient, la proportion de cas de poliomyélite attribuables à des VDPV augmentait. En 2016 pour la première fois, le nombre de cas de poliomyélite dus à des VDPV a excédé celui des cas dus aux virus sauvages.

Alors qu'aucun virus sauvage de type 2 n'a été détecté depuis 1999, plus de 90% des cas de poliomyélite dus à des VDPV depuis leur découverte en 2000 étaient causés par des VDPV de type 2 (VDPV-2). La décision a donc été prise de ne plus utiliser la souche vaccinale Sabin 2 et de passer du VPO trivalent historique à un VPO bivalent ne contenant que les souches Sabin 1 et 3 [9]. Mondialement synchronisé, le passage de l'un à l'autre a eu lieu en avril 2016 [7, 24]. Cette stratégie comporte toutefois un risque car, dans les pays où seul le VPO est utilisé, aucun enfant né après cette date n'est immunisé contre le sérotype 2. Afin de faire face à l’éventuelle résurgence de VDPV-2, un stock de VPO monovalent ne contenant que la souche Sabin 2 a été constitué. Celui-ci ne devait en théorie être utilisé qu'avec parcimonie car, dans un monde où de nombreux enfants nés après l'abandon du VPO trivalent ne sont plus immunisés contre le type 2, le risque de voir essaimer la souche atténuée qu'il contient était grand. Le scénario redouté s'est malheureusement déroulé en Afrique: en République démocratique du Congo, plusieurs cas de poliomyélite ont été attribués en 2017 et 2018 à des VDPV-2 probablement dérivés de souches vaccinales administrées avant l'abandon du VPO trivalent [15]. Pour stopper la circulation de ces VDPV-2, l'OMS a dû recourir à l'utilisation du vaccin monovalent de type 2. Or la souche Sabin 2 qu'il contient a pu diffuser dans les pays voisins où seul le vaccin bivalent immunisant contre les types 1 et 3 était utilisé. De nouveaux VDPV-2 ont alors émergé dans ces pays, nécessitant l'utilisation de nouvelles doses de VPO monovalent. De proche en proche, de nombreux pays d'Afrique subsaharienne, du Sénégal à la Somalie, ont connu l’émergence de VDPV-2 depuis l'abandon du VPO trivalent [2]. Des VDPV-2 ont également émergé durant cette période dans la région composée du Pakistan, de l'Afghanistan et du Tadjikistan. En compromettant la qualité de la surveillance et en compliquant la mise en place de ripostes vaccinales, la crise sanitaire due à la Covid-19 a facilité ces émergences.

Le programme d’éradication s'est alors trouvé pris dans un cercle vicieux: l'extinction des épidémies de VDPV-2 requiert l'utilisation de la souche Sabin 2 alors que celle-ci constitue la source de ces épidémies. Deux stratégies étaient envisageables pour sortir de cette situation. La première consiste à réintroduire la souche Sabin 2 dans le vaccin de routine et à atteindre une couverture vaccinale suffisamment élevée à l’échelle mondiale pour empêcher la circulation de cette souche et interdire ainsi l’émergence de VDPV-2. Cette stratégie semble illusoire puisque c'est précisément l'impossibilité d'atteindre une couverture élevée dans certaines régions du monde qui a conduit à abandonner la souche Sabin 2. La deuxième stratégie consiste à développer une nouvelle souche vaccinale de type 2: à l'instar de la souche Sabin 2, elle doit être capable d'induire une réponse immunitaire intestinale mais ne pas pouvoir recouvrer un phénotype neurovirulent, même en cas de circulation au sein d'une population insuffisamment immunisée.

## Une nouvelle souche vaccinale

Une nouvelle souche vaccinale (S2/S15domV/cre5/rec1/hifi3) a été conçue par génie génétique à partir du génome de la souche Sabin 2 [25]. L'objectif était de stabiliser la conformation du domaine V de la région non codante 5’ à l'origine du phénotype atténué. Plusieurs mutations ont été introduites dans ce domaine afin que la réversion d'une seule position nucléotidique ne suffise pas à restaurer la capacité du virus à se répliquer dans des cellules nerveuses (Fig. 3). Afin d’éviter que la région 5’ non codante responsable de l'atténuation ne soit substituée par une autre au cours d'un évènement de recombinaison avec un entérovirus non polio, une courte région indispensable à la réplication du génome a été déplacée. Cette séquence (appelée *cre* pour *cis-replicating element)* se situe normalement dans la deuxième moitié du génome viral. Une copie de la séquence *cre* a été introduite au sein de la région 5’ non codante de la nouvelle souche vaccinale tandis que la fonctionnalité de la séquence *cre* native a été abolie par une série de mutations. Les événements de recombinaison entre entérovirus entraînant généralement la substitution de la région 5’ non codante entière, la recombinaison de la nouvelle souche vaccinale avec un autre entérovirus au niveau de cette région donnerait naissance à un génome rendu non fonctionnel par l'absence de séquence cre. La nouvelle souche contient également deux mutations ponctuelles au sein de la région codant la polymérase virale, une enzyme responsable de la réplication du génome viral: l'une augmente la fidélité de la polymérase (et donc diminue le risque d'incorporer des mutations lors de la réplication du génome) tandis que l'autre diminue la fréquence de recombinaison.

La nouvelle souche vaccinale a tout d'abord été étudiée *in vitro* [25]. En premier lieu, il a été vérifié que le virus issu du génome modifié par génie génétique se multipliait aussi bien dans des cultures cellulaires que la souche Sabin 2. Une souche présentant un pouvoir réplicatif trop faible pourrait en effet entraîner une réponse immunitaire insuffisante. La stabilité des mutations incorporées dans le génome a également été vérifiée par des passages successifs du virus en cultures cellulaires. Enfin, l'atténuation du virus, même après de nombreux passages en cultures cellulaires, a pu être vérifiée dans un modèle murin.

L'immunogénicité et la stabilité génétique de la nouvelle souche vaccinale ont été testées par administration à un groupe de 15 volontaires [22]. Afin de prévenir tout risque de survenue de poliomyélite en cas de réversion, tous les participants avaient été vaccinés avec le VPI. Pour éviter la dissémination de la nouvelle souche vaccinale dans l'environnement, les volontaires ont été confinés durant quatre semaines au sein d'un bâtiment spécialement construit en Belgique. Ce bâtiment a été conçu afin de collecter tous les déchets et les effluents liquides en vue de leur décontamination [23]. Aucun effet indésirable sévère n'a été observé parmi les volontaires. Tous ont excrété la souche vaccinale durant au moins une semaine avec une durée maximale d'excrétion de 89 jours chez l'un des volontaires. L'utilisation d'un modèle murin a permis de montrer que les virus excrétés n’étaient pas neurovirulents tandis que le séquençage de leur génome ne montrait aucune réversion au niveau des nucléotides modifiés. Enfin, une augmentation du titre d'anticorps neutralisants dirigés contre le sérotype 2 a été observée chez quasiment tous les volontaires. Des essais cliniques impliquant quelques centaines de volontaires préalablement vaccinés avec le VPO ou le VPI ont ensuite confirmé la bonne tolérance de la nouvelle souche vaccinale et son immunogénicité [8, 21].

Les résultats obtenus *in vitro* et lors des essais cliniques ont conduit le Groupe stratégique consultatif d'experts sur la vaccination à approuver l'utilisation de la nouvelle souche atténuée dans le cadre du protocole EUL (pour Emergency Use Listing). Ce protocole permet à l'OMS d'autoriser l'utilisation de vaccins, de médicaments ou de tests de diagnostic non homologués en réponse à une situation d'urgence. L'autorisation délivrée par l'OMS le 13 novembre 2020 s'accompagne de la définition d'un cadre strict qui prévoit notamment une surveillance renforcée de la circulation des poliovirus et le recueil des effets indésirables associés à la vaccination par la nouvelle souche vaccinale.

Pour l'heure, une seule compagnie fabrique le nouveau vaccin (Bio Farma en Indonésie). Plus d'une vingtaine de pays ont entamé les démarches auprès de l'OMS pour pouvoir mettre en œuvre le nouveau vaccin en réponse à des épidémies de VDPV-2. Le Nigéria, le Libéria, le Bénin, la Sierra Leone et le Tadjikistan sont les premiers à l'avoir effectivement utilisé au cours de campagnes organisées à partir du deuxième trimestre 2021.

La collecte des données concernant l'effet de la nouvelle souche vaccinale sur la circulation des VDPV-2 existants est en cours. Si l'immunogénicité de la nouvelle souche vaccinale ne laisse aucun doute, sa capacité à limiter l’émergence de VDPV-2 reste à déterminer. Malgré les modifications apportées à son génome, il existe un scénario qui permettrait l’émergence de VDPV-2 pathogènes à partir de la nouvelle souche vaccinale: celui d'une succession d’évènements de recombinaison avec des entérovirus non polio dont l'un permettrait d'abord au génome de la souche vaccinale de recouvrer une séquence *cre* fonctionnelle à sa localisation normale avant qu'un deuxième n'entraîne la perte du domaine V modifié. Une intense surveillance de la circulation de la nouvelle souche vaccinale et de l’évolution de son génome est effectuée en collectant des eaux usées dans les pays qui utilisent le nouveau vaccin afin d’évaluer le risque d’émergence de VDPV-2 à partir de la nouvelle souche vaccinale.

## Conclusion

Le programme d’éradication polio a obtenu d'indéniables succès: éradication de deux des trois sérotypes sauvages, réduction drastique de l'aire de circulation des virus et du nombre annuel de cas. Il est aujourd'hui confronté à deux défis: éradiquer le dernier sérotype sauvage qui subsiste encore au Pakistan et en Afghanistan, et prévenir l’émergence de VDPV.

Le premier défi est dû à la situation politique des deux pays qui cumulent de nombreuses difficultés: le manque d'influence du pouvoir central dans certaines régions, les difficultés logistiques, l'insécurité, le rejet de la vaccination, les attaques ciblant les équipes de vaccination et les attentats suicides dans les centres de vaccination compromettent la mise en place de campagnes exhaustives [12]. L'exemple africain permet néanmoins d’être optimiste puisque le continent a été déclaré exempt de poliovirus sauvages l'an dernier [14]: la dernière région où circulaient encore les virus sauvages était le Nigéria où la situation présentait de nombreux traits communs avec celle du Pakistan et de l'Afghanistan [1, 18].

Le problème que posent aujourd'hui les VDPV nous rappelle que la pertinence des outils mis en œuvre dans le cadre de programmes de santé publique n'est pas une propriété intrinsèque mais varie en fonction du contexte et avec le temps. Le VPO d'Albert Sabin était un outil adapté à l’époque où les poliovirus sauvages circulaient activement sur tout le globe et causaient des centaines de milliers de cas de poliomyélite annuellement; alors que ce nombre de cas est de l'ordre de quelques centaines, le VPO est aujourd'hui obsolète. La modification par génie génétique de la souche vaccinale historique offre une nouvelle solution prometteuse. La conception de cette souche contraste avec celle des souches atténuées historiques qui relevait d'une démarche empirique. Elle a été rendue possible par l'immense travail de recherche fondamentale qui a été effectué durant des décennies sur les poliovirus et qui a permis d'expliquer les mécanismes sous-tendant l'atténuation, de comprendre les processus à l'origine de la réversion et d'identifier des mutations ponctuelles associées à une fréquence de réversion moindre. Dans le cas où les modifications apportées à Sabin 2 pour obtenir la nouvelle souche vaccinale se révéleraient insuffisantes pour prévenir l’émergence de VDPV-2, des modifications génétiques supplémentaires pourraient y être introduites afin de constituer des freins additionnels à la réversion [13].

Si cette stratégie fonctionne, elle sera appliquée à la conception de nouvelles souches atténuées des sérotypes 1 et 3 qui remplaceront les souches Sabin 1 et 3. De telles souches, construites sur le modèle de la nouvelle souche vaccinale de type 2, ont déjà été produites et sont en cours d’évaluation (https://clinicaltrials.gov/ct2/show/NCT04529538?term=nopv&draw=2&rank=6).

## Lien d'intérêts

L'auteur ne déclare aucun lien d'intérêt.
